# Allergic Contact Dermatitis Associated With Hydroxyacetophenone: Two Case Reports

**DOI:** 10.1111/cod.70123

**Published:** 2026-03-07

**Authors:** Samia Leghlam, Evelyne Collet, Louise Collin, Camille Leleu

**Affiliations:** ^1^ Dermato‐Allergology Department CHU Dijon Bourgogne Dijon France

**Keywords:** allergic contact dermatitis, CAS 99–93‐4, Hydroxyacetophenone

Hydroxyacetophenone (CAS 99–93‐4) is a phenolic compound found in Norway spruce and widely used in cosmetics for its antioxidant properties [1]. This report adds two more cases linked to cosmetic products containing hydroxyacetophenone. Oral informed consent was obtained from the patients for the publication of their images and clinical details.

## 1 Case Reports

A 63‐year‐old male, with a history of psoriasis, attended for a follow‐up consultation. His topical treatment was adjusted to include a psoriasis‐specific emollient cream (XEMOSE PSO, Uriage) and topical steroids.

**FIGURE 1:**
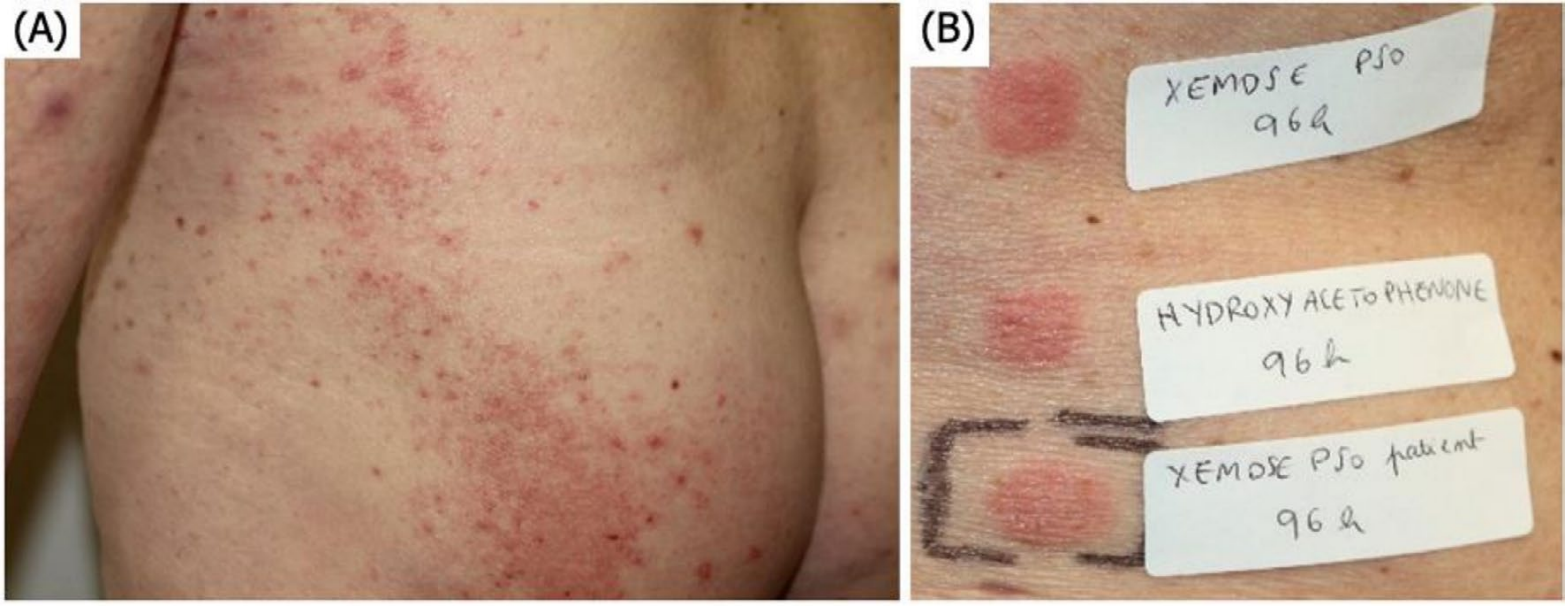
(A) Clinical presentation and (B) patch‐test reading at D4.

Seven days later, the patient developed eczematous plaques on the flanks, buttocks, and eyelids (Figure 1A). A skin biopsy showed eczematous dermatitis with no abnormalities on direct immunofluorescence.

The repeated application test (ROAT) was positive for XEMOSE PSO. Patch‐tests (Chemotechnique Diagnostics, Vellinge, Sweden) with the ingredients of the emollient cream confirmed a strong positivity (++) for hydroxyacetophenone provided by the manufacturer (0,6% aq) at D2 and D4 (Figure 1B).

Discontinuation of the implicated products and local treatments led to a gradual improvement of the eczema.

**FIGURE 2:**
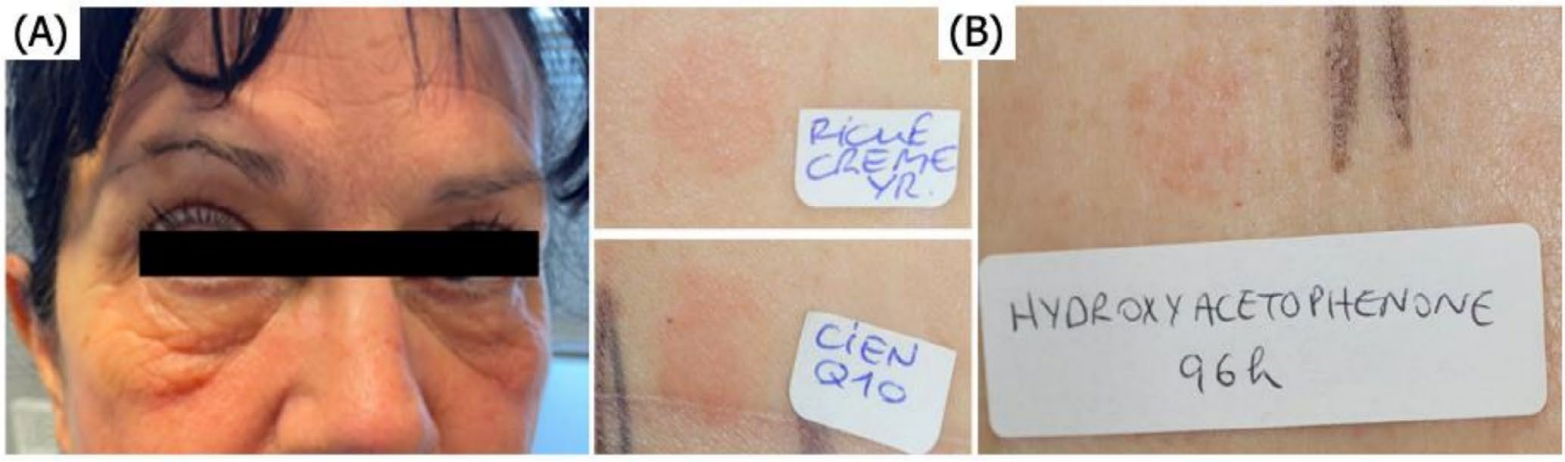
(A) Clinical presentation and (B) patch‐test reading at D4.

A 66‐year‐old female presented with a 6‐month history of periorbital edema of the lower lid and facial eczema (Figure 2 A). She reported the daily use of an anti‐wrinkle day cream (Crème Riche, Yves Rocher, France), a night cream (Cien Q10, Lidl), a serum, and sunscreen.

Patch‐testing was performed using the European Baseline Series, the additional allergens from the GERDA series, and the specific products used by the patient. The patient had strong positive reactions (++) at D4 to both anti‐wrinkle creams and to hydroxyacetophenone (0,6% aq), a common ingredient to both (Figure 2 B). A mild response (+) was already observed at D2

## 2 Discussion

Hydroxyacetophenone is not widely recognised as a contact allergen. Nonetheless, three cases of contact dermatitis associated with this compound and one case of photo‐aggravated allergic contact dermatitis have been documented. The first case involved occupational eczema in a paper mill worker exposed to a myxobactericide containing 2‐bromo‐4′‐hydroxyacetophenone. Patch testing confirmed sensitization to hydroxyacetophenone [2]. In the second case, a woman developed pruritic facial dermatitis following the use of a cosmetic cream containing hydroxyacetophenone, with patch testing confirming the allergic reaction and resolution upon discontinuation of the product [3]. Similarly, the third case described facial eczema in a patient using an anti‐wrinkle serum, with hydroxyacetophenone identified as the sensitising agent through patch testing [4]. More recently, Pesqué et al. described a case of corticosteroid‐resistant facial and cervical eczema associated with the use of a sunscreen containing hydroxyacetophenone. Patch testing with hydroxyacetophenone (0.6% aq) resulted in a mild (+) reaction, while photopatch testing produced a stronger (++) response. This case highlights the need to consider hydroxyacetophenone as a potential photoallergen [5]. To date, no standardised concentration for patch testing hydroxyacetophenone has been established. Nevertheless, testing at 0.6% in water appears sufficient to detect sensitization.

Additionally, several cases of allergic contact dermatitis related to resacetophenone, a compound used in nail antifungal preparations, have been documented. However, the potential for cross‐reactivity between hydroxyacetophenone and resacetophenone remains unknown [6, 7, 8].

We present two new cases of contact dermatitis caused by hydroxyacetophenone, a widely used preservative in cosmetics and industrial products. These findings suggest that hydroxyacetophenone should be considered as a potential allergen in individuals presenting with contact dermatitis, especially in those using cosmetic formulations containing this preservative.

## Author Contributions

**Samia Leghlam:** writing – original draft. **Evelyne Collet:** supervision. **Louise Collin:** supervision. **Camille Leleu:** writing – review and editing.

## Conflicts of Interest

The authors declare no conflicts of interest.

## Data Availability

The data that support the findings of this study are available from the corresponding author upon reasonable request.
